# Supplementary material for: Chalcones as Anti-Glioblastoma Stem Cell Agent Alone or as Nanoparticle Formulation Using Carbon Dots as Nanocarrier
Source: Pharmaceutics. 2022 Jul 14;14(7):1465. doi: 10.3390/pharmaceutics14071465 (PMC9316063; doi:10.3390/pharmaceutics14071465)
Supplement: Supplementary file 1 [file pharmaceutics-14-01465-s001.zip › pharmaceutics-1789542-supplementary.pdf]

Supporting Information

Experimental Methods Section

General: all reagents were obtained from commercial sources and were used without further purification unless noted otherwise. Thin layer chromatography (TLC) was performed with Merck silica gel 60 F254 pre-coated plates, eluting with the solvents indicated. Short and long wave visualization was performed with a Mineralight multiband ultraviolet lamp at 254 and 365 nm, respectively. ^{1}H and ^{13}C Nuclear Magnetic Resonance spectra of pure compounds were recorded either on AVANCE II 400 or AVANCE 500 (Bruker). Chemical shifts are reported in parts per million in reference to the solvent peak. The abbreviations s, d, dd, t, td, q, m, and brs stand for singlet, doublet, doublet of doublets, triplet, triplet of doublets, quartet, multiplet, and broad singlet, in that order. High resolution mass spectra were obtained on a Bruker micrOTO-Q II spectrometer.

General procedure for the synthesis of 4'-amino chalcones

Method A: to a stirred solution of substituted benzaldehyde (1.1 equiv.) and 4'-aminoacetophenone (1 equiv.) in methanol (5 mL) was added a 1:1 mixture of methanol and 20% aq. NaOH (2 mL). The reaction mixture was stirred at room temperature for 24-48 h. The reaction mixture was diluted with water and then refrigerated overnight. The solid obtained was collected by vacuum filtration. The purity of the crude product was determined by TLC analysis and NMR spectroscopy. If needed, the solid was either purified by recrystallization from methanol or by flash column chromatography (FCC) using EtOAc/hexanes as solvent to give the analytically pure product.

Method B: to a stirred solution of the substituted benzaldehyde (1.1 equiv.) and 4'-aminoacetophenone (1 equiv.) in methanol (6 mL) was added thionyl chloride (5 drops). The mixture was heated under gentle reflux for 24 h. The reaction mixture was cooled down, concentrated to 1/3 of its volume, and diluted with water. The solid obtained was collected by vacuum filtration. The purity of the crude product was determined by TLC analysis and NMR spectroscopy. . If needed, the solid was either purified by recrystallization from methanol or by flash column chromatography (FCC) using EtOAc/hexanes as solvent to give the analytically pure product.

1-(4-aminophenyl)-3-(3-ethoxy-4-hydroxyphenyl)prop-2-en-1-one (**3j**): the general procedure B was followed to give the product as a red solid. Recrystallization afforded the product in 35% yield; ^{1}H NMR (500 MHz, DMSO-d$_6$) δ (ppm): 9.46 (br s, 1H), 7.92 (d, 2H, J = 8.5 Hz), 7.67 (d, 1H, J = 15.5 Hz), 7.54 (d, 1H, J = 15.5 Hz), 7.44 (s, 1H), 7.20 (d, 1H, J = 8 Hz), 6.83 (d, 1H, J = 8 Hz), 6.63 (d, 2H, J = 8.5 Hz). 6.07 (s, 2H), 4.13 (q, 2H, J = 7 Hz), 1.37 (t, 3H, J = 7 Hz); ^{13}C NMR (125 MHz , DMSO-d$_6$) δ (ppm): 186.4, 154.1, 149.8, 147.6, 142.8, 131.4, 127,2, 126.2, 123.9, 119.5, 116.1, 113.2, 64.5, 15.2. HRMS calculated for $[M+1]^+$ 283.1208, found 283.1292.

1-(4-Aminophenyl)-3-(3-methoxy-4-methoxymethoxyphenyl)prop-2-en-1-one (**3h**): the general procedure A was followed. Purification by FCC (EtOAc/hexanes 1:1) gave the product as a yellow solid with the isolate yield 46%; ^{1}H NMR (400 MHz, CD_2Cl_2) δ (ppm): 7.93 (d, 2H, J = 8.4 Hz), 7.71 (d, 1H, J = 15.6 Hz), 7.49 (d, 1H, J = 15.6 Hz), 7.25 (d, 2H, J = 1.6 Hz), 7.23 (s, 2H), 7.15 (d, 1H, J = 8.8 Hz), 6.74 (d, 2H, J = 8.8 Hz), 5.25 (s, 2H), 4.27 (br s, 2H), 3.94 (s, 3H), 3.52 (s, 3H); ^{13}C NMR (100 MHz , CD_2Cl_2) δ (ppm): 187.4, 151.5, 150.2, 148.5, 142.6, 130.8,

129.8, 128.4, 122.1, 120.5, 116.4, 113.7, 111.1, 95.4, 56.2, 55.9. HRMS calculated for $[M+1]^+$ 314.1314, found 314.1390.

1-(4-Aminophenyl)-3-[4-(4-morpholinyl)phenyl]prop-2-en-1-one (**3m**): the general procedure A was followed. Purification by FCC (EtOAc/hexanes 1:1) gave the product as yellow solid with the isolate yield 75%; ^{1}H NMR (500 MHz, CD_2Cl_2) δ (ppm): 7.93 (d, 2H, *J* = 8.5 Hz), 7.73 (d, 1H, *J* = 15.5 Hz), 7.61 (d, 2H, *J* = 8.5 Hz), 7.46 (d, 1H, *J* = 15.5 Hz), 6.94 (d, 2H, J = 8.5 Hz), 6.74 (d, 2H, *J* = 8.5 Hz), 4.26 (s, 2H), 3.86 (t, 4H, *J* = 5 Hz), 3.28 (t, 4H, *J* = 4.5 Hz; ^{13}C NMR (125 MHz, CD_2Cl_2) δ (ppm): 188.4, 160.1, 153.2, 144.6, 138.4, 136.5, 130.0, 125.7, 121.1, 121.0, 116.5, 113.7, 55.3. HRMS calculated for $[M+1]^+$ 309.1525, found 309.1603.

General procedure for the synthesis of *N*-acetyl chalcones (3d and 3g) and *O*-acetyl chalcone (4d)

To a stirred solution of the chalcone (1 equiv.) and triethylamine (3 equiv.) in DCM (3 mL) was added acetyl chloride (2 equiv.). The reaction mixture was stirred at room temperature for 24 h. Then, aq. sat. $NaHCO_3$ solution (5 mL) was added to the mixture and stirred for 10 minutes. The organic layer was separated, washed with brine, and dried over anh. Na_2SO_4. Filtration and then evaporation afforded the crude product. The acetylated product was purified by FCC using EtOAc/hexanes (1:1) as solvent.

4-(3-(2,5-Dimethoxyphenyl)propenoyl) acetanilide (**3d**): the pure product was obtained as a yellow solid in 55% yield. ^{1}H NMR (500 MHz, $CDCl_3$) δ (ppm): 8.09 (d, 1H, *J* = 16 Hz), 8.03 (d, 2H, *J* = 8.5 Hz), 7.68 (d, 2H. *J* = 8.5 Hz), 7.64 (br s, 1H), 7.60 (d, 1H, *J* = 16 Hz), 7.18 (d, 1H, *J* = 3 Hz), 6.96 (dd, 1 H, *J* = 3 Hz, *J* = 9 Hz), 6.89 (d, 1H, *J* = 9 Hz), 3.88 (s, 3H), 3.84 (s,

3H), 2.24 (s, 3H); ^{13}C NMR (125 MHz, $CDCl_3$) δ (ppm): 189.8, 168.8, 153.5, 153.4, 142.2, 140.0, 133.9, 130.0, 124.5, 122.8, 119.0, 117.3, 113.8, 112.5, 56.1, 55.9, 24.8. HRMS calculated for $[M+1]^+$ 326.1314, found 326.1399.

4-(3-(3,5-Dimethoxyphenyl)propenoyl) acetanilide (**3g**): the pure product was obtained as a pale yellow solid in 95% yield. ^1H NMR (500 MHz, $CDCl_3$) δ (ppm): 8.03 (d, 2H, *J* = 8.5 Hz), 7.72 (d, 1H, *J* = 15.5 Hz), 7.64 (d, 2H, *J* = 8 Hz), 7.59 (br s, 1H), 7.48 (d, 1H, *J* = 16 Hz), 7.79 (d, 2H, *J* = 2.5 Hz), 6.54 (t, 1H, *J* = 2.5 Hz), 3.86 (s, 6H), 2.24 (s, 3H); ^{13}C NMR (125 MHz, $CDCl_3$) δ (ppm): 189.1, 161.1, 144.6, 142.1, 136.8, 130.0, 122.3, 119.0, 106.4, 102.8, 55.5. HRMS calculated for $[M+1]^+$ 326.1314, found 326.1400.

Figure S1. ^{1}H and ^{13}C Nuclear Magnetic Resonance spectra for synthesized compounds

SpinWorks 4: 1-(4-Aminophenyl)-3-(4-hydroxy-3-ethoxyphenyl)prop-2-en-1-one

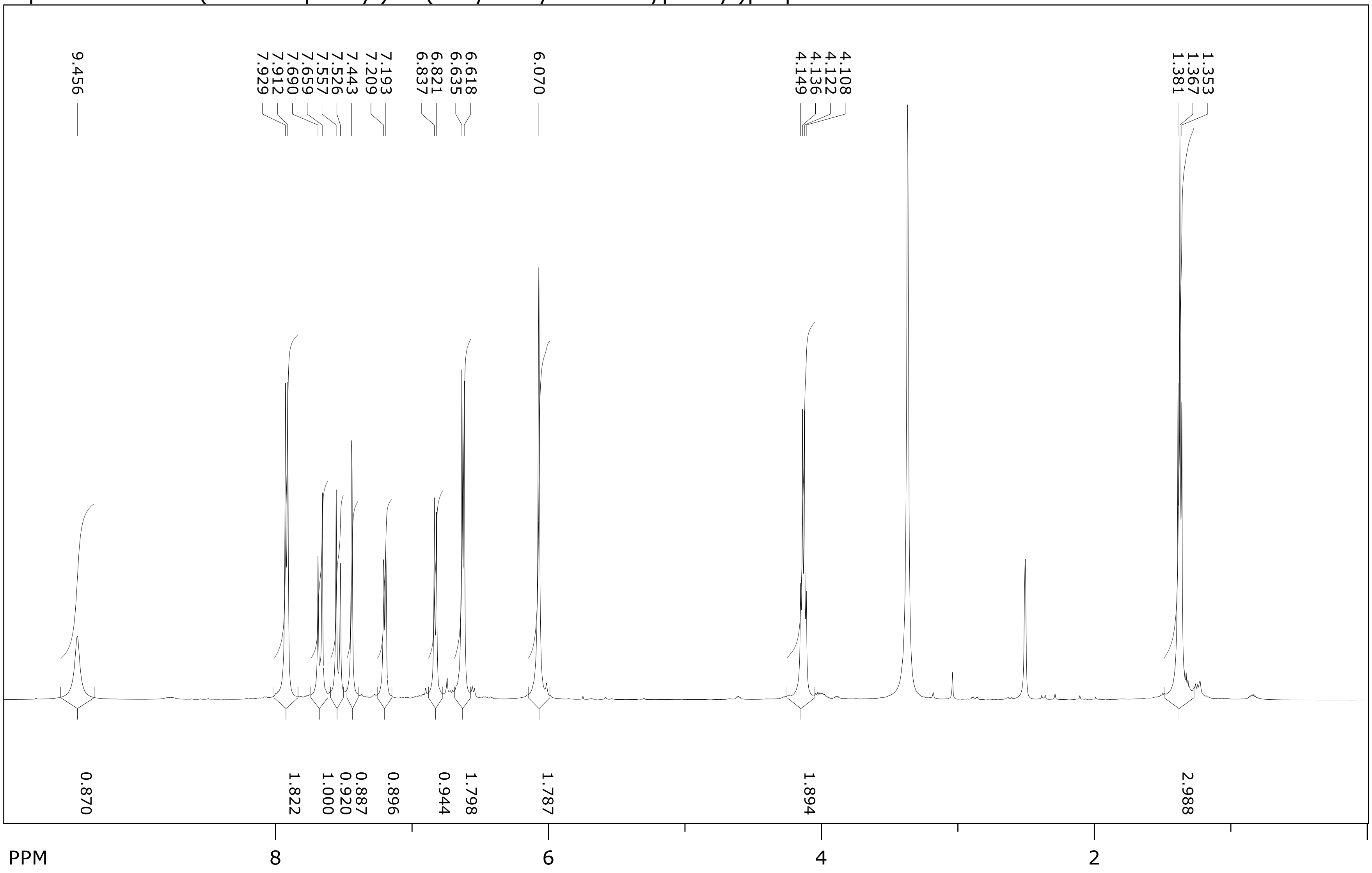

file: ... Data\EAV\500NMR\EAV-VIII-59\1\fid expt: <zg30>
transmitter freq.: 500.063088 MHz
time domain size: 65536 points
width: 7507.51 Hz = 15.0131 ppm = 0.114555 Hz/pt
number of scans: 32

freq. of 0 ppm: 500.060000 MHz
processed size: 32768 complex points
LB: 0.300 GF: 0.0000

SpinWorks 4: 1-(4-Aminophenyl)-3-(4-hydroxy-3-ethoxyphenyl)prop-2-en-1-one

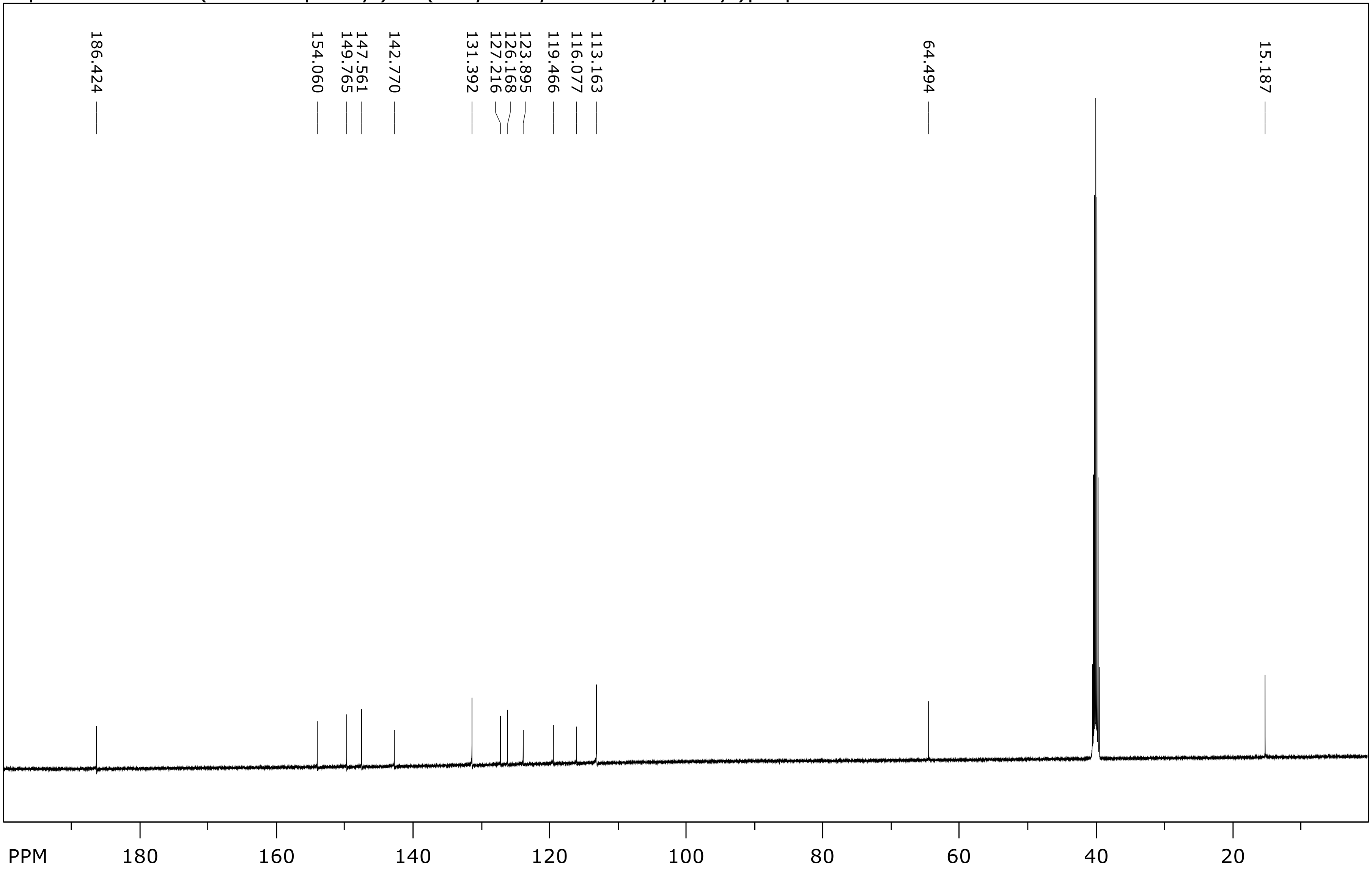

file: ...Data\EAV\500NMR\EAV-VIII-59C\1\fid expt: <zgpg>
transmitter freq.: 125.752761 MHz
time domain size: 262144 points
width: 26455.03 Hz = 210.3733 ppm = 0.100918 Hz/pt
number of scans: 1416

freq. of 0 ppm: 125.740187 MHz
processed size: 131072 complex points
LB: 0.300 GF: 0.0000

SpinWorks 4: 1-(4-Aminophenyl)-3-(3-methoxy-4-methoxymethoxyphenyl)prop-2-en-1-one

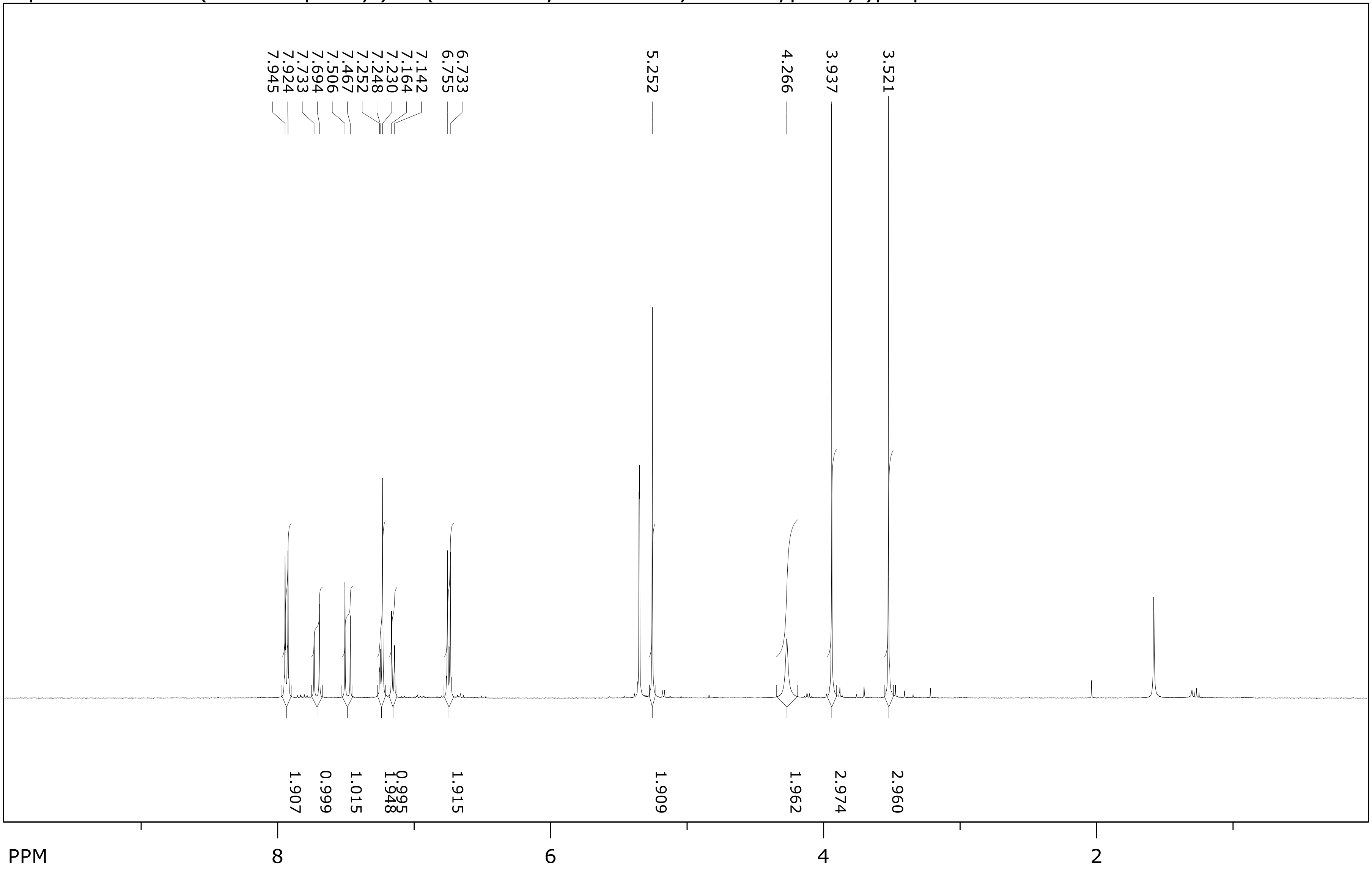

file: ...MR Data\EAV\400NMR\EAV-X-138\1\fid expt: <zg30>
transmitter freq.: 399.995470 MHz
time domain size: 65536 points
width: 8278.15 Hz = 20.6956 ppm = 0.126314 Hz/pt
number of scans: 16

freq. of 0 ppm: 399.993000 MHz
processed size: 32768 complex points
LB: 0.300 GF: 0.0000

SpinWorks 4: 1-(4-Aminophenyl)-3-(3-methoxy-4-methoxymethoxyphenyl)prop-2-en-1-one

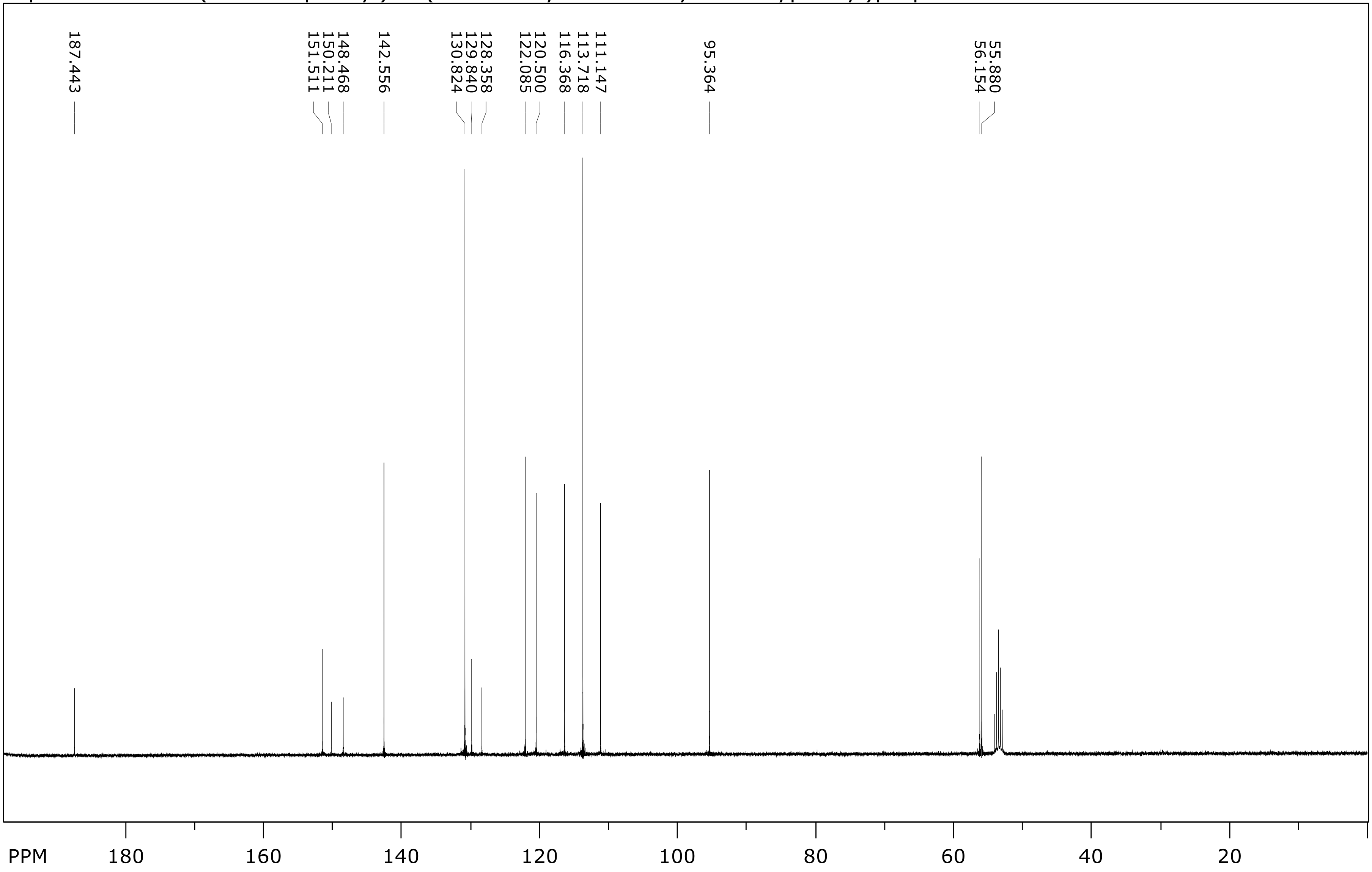

file: ...R Data\EAV\400NMR\EAV-X-138C\1\fid expt: <zgpg>
transmitter freq.: 100.586869 MHz
time domain size: 32768 points
width: 22675.74 Hz = 225.4344 ppm = 0.692009 Hz/pt
number of scans: 1907

freq. of 0 ppm: 100.578320 MHz
processed size: 131072 complex points
LB: 0.300 GF: 0.0000

SpinWorks 4: 1-(4-Aminophenyl)-3-[4-(4-morpholinyl)phenyl]prop-2-en-1-one

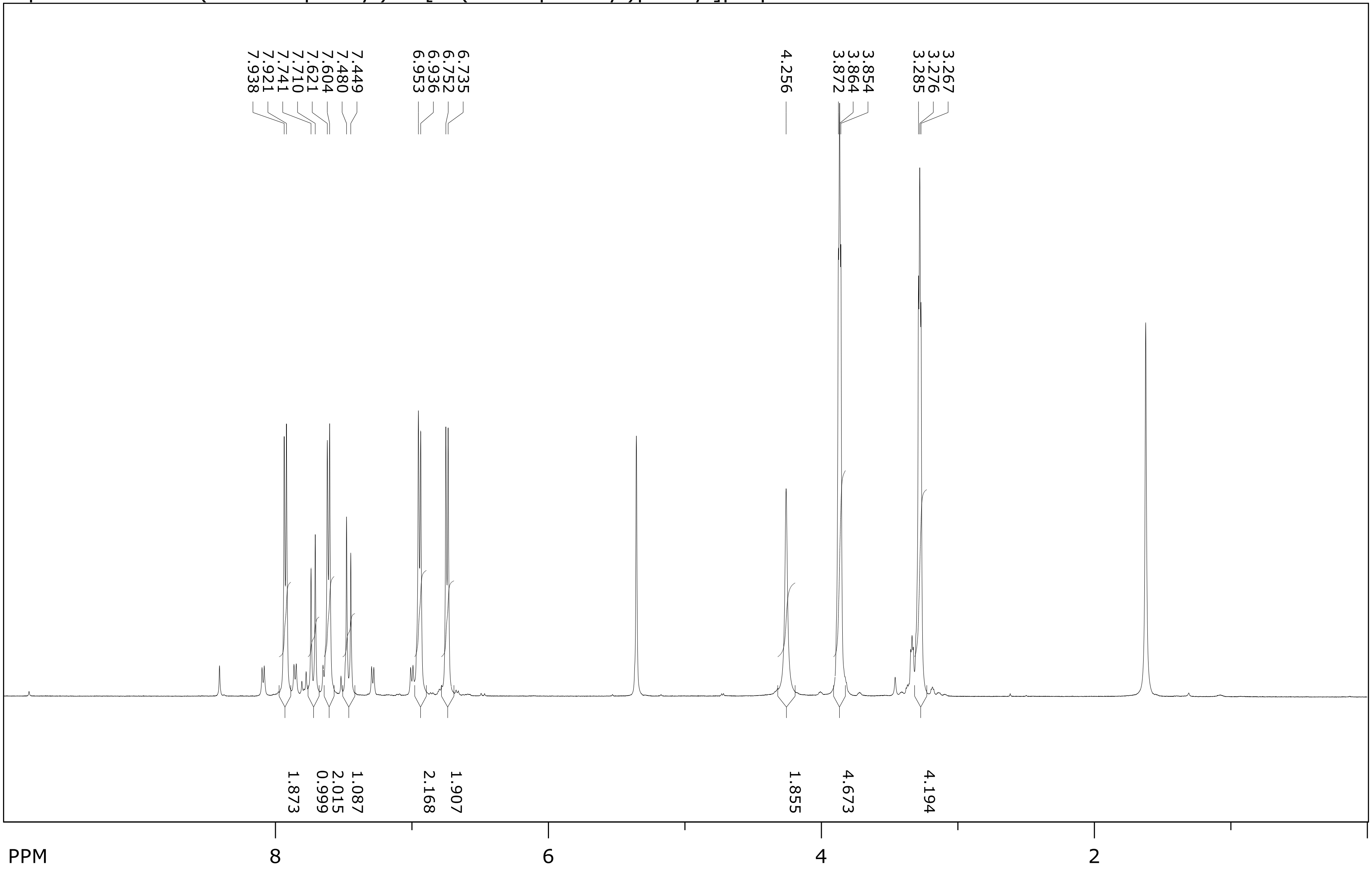

file: ...MR Data\EAV\500NMR\EAV-X-130\1\fid expt: <zg30>
transmitter freq.: 500.063088 MHz
time domain size: 32766 points
width: 7507.51 Hz = 15.0131 ppm = 0.229125 Hz/pt
number of scans: 2

freq. of 0 ppm: 500.060000 MHz
processed size: 32768 complex points
LB: 0.300 GF: 0.0000

SpinWorks 4: 4-(3-(2,5-Dimethoxyphenyl)propenoyl) acetanilide

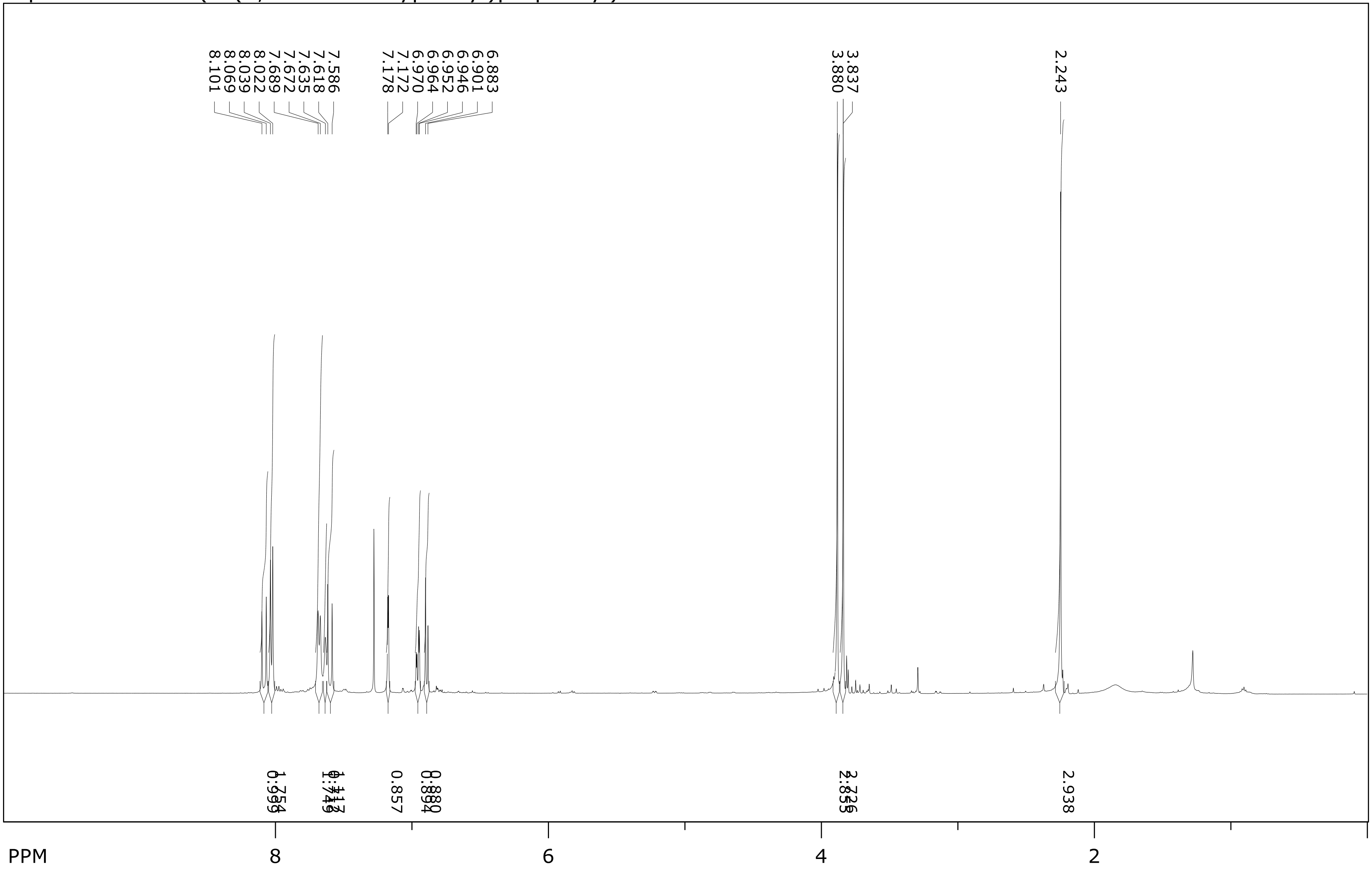

file: ...Data\EAV\500NMR\EAV-VIII-119\1\fid expt: <zg30>
transmitter freq.: 500.063088 MHz
time domain size: 40238 points
width: 7507.51 Hz = 15.0131 ppm = 0.186578 Hz/pt
number of scans: 8

freq. of 0 ppm: 500.060000 MHz
processed size: 32768 complex points
LB: 0.300 GF: 0.0000

SpinWorks 4: 4-(3-(2,5-Dimethoxyphenyl)propenoyl) acetanilide

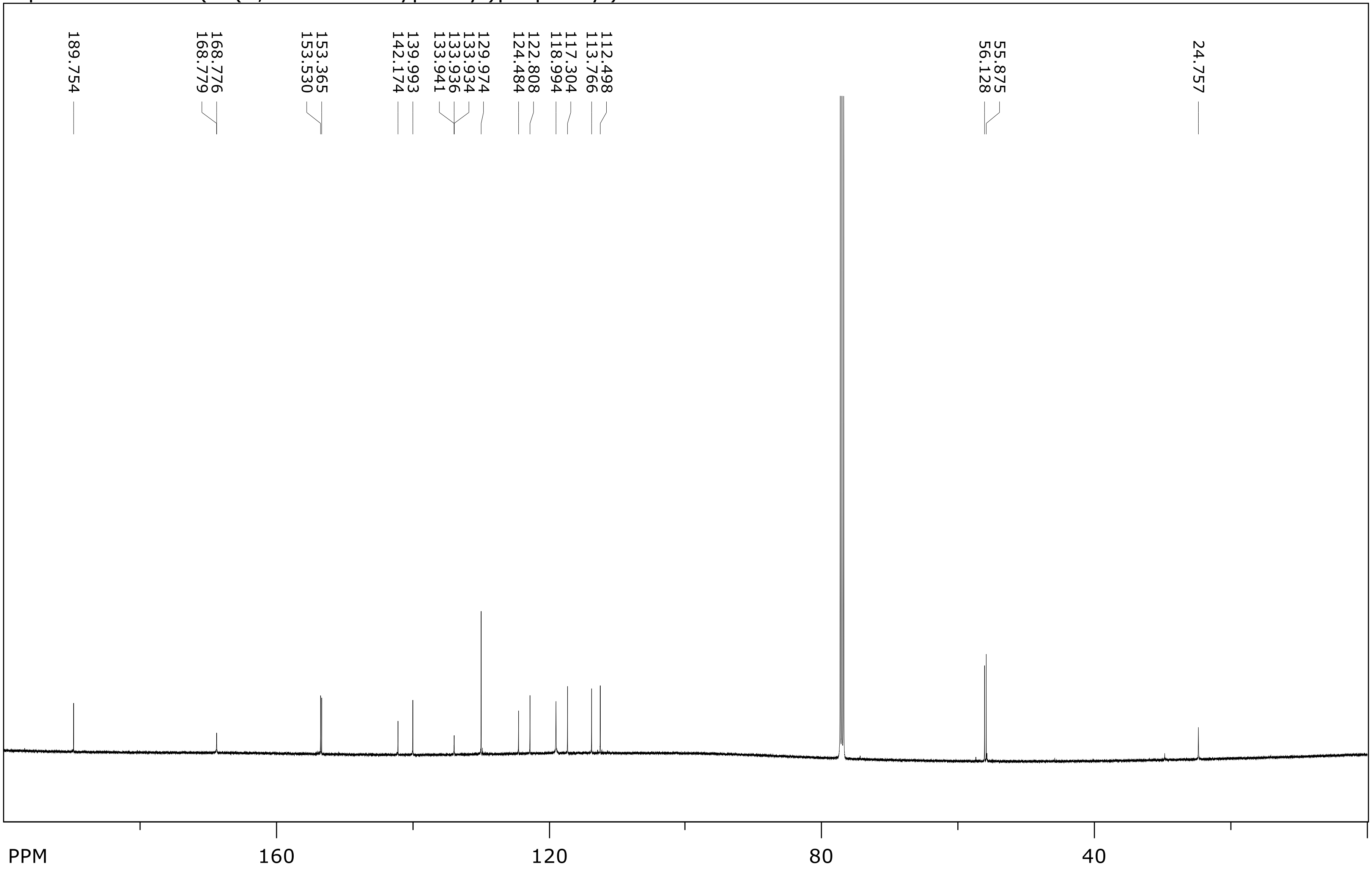

file: ...ata\EAV\500NMR\EAV-VIII-119C\1\fid expt: <zgpg>
transmitter freq.: 125.752761 MHz
time domain size: 137562 points
width: 26455.03 Hz = 210.3733 ppm = 0.192313 Hz/pt
number of scans: 2000

freq. of 0 ppm: 125.740187 MHz
processed size: 131072 complex points
LB: 0.300 GF: 0.0000

SpinWorks 4: 4-(3-(3,5-Dimethoxyphenyl)propenoyl) acetanilide

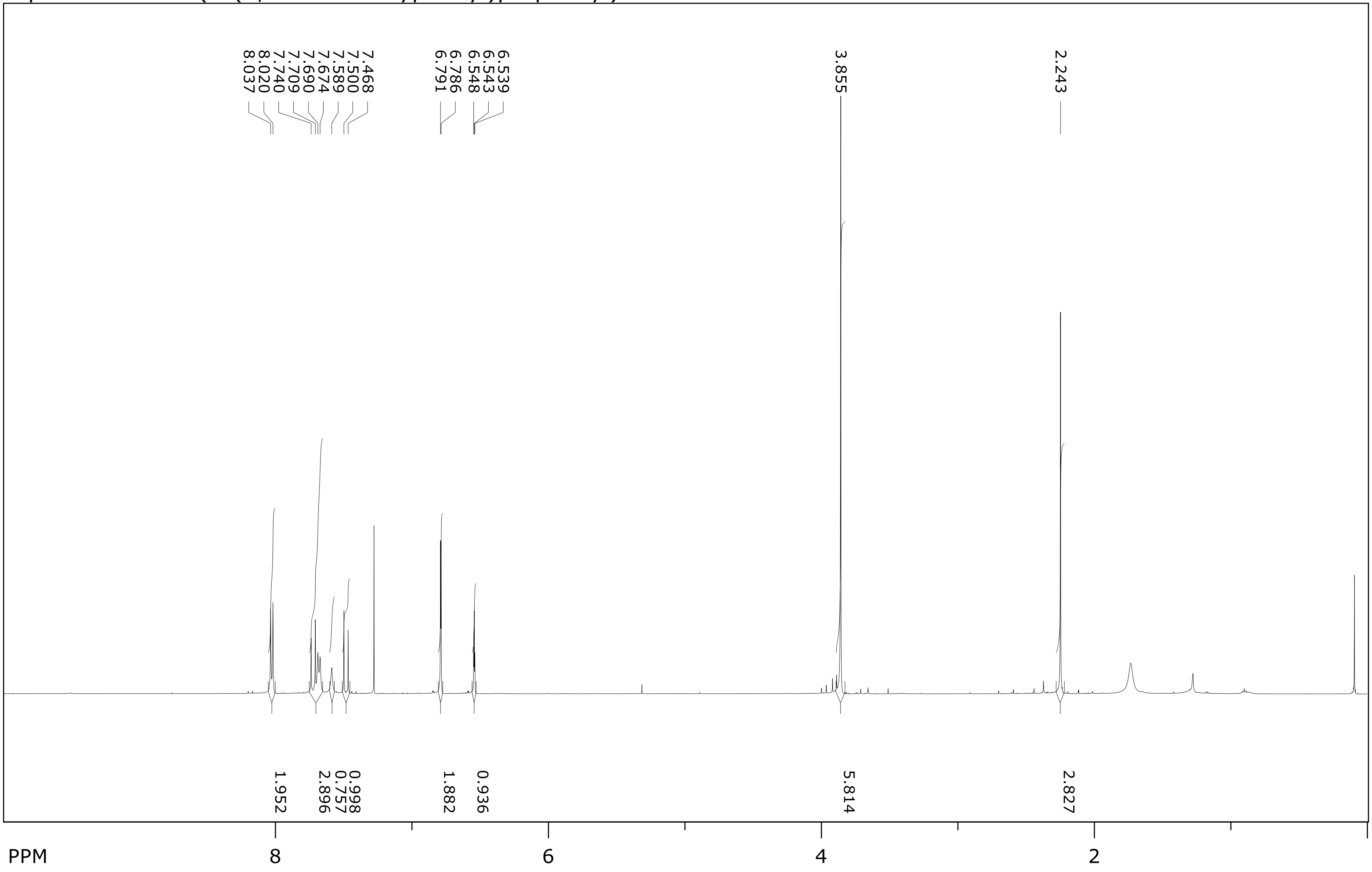

file: ...Data\EAV\500NMR\EAV-VIII-77A\1\fid expt: <zg30>
transmitter freq.: 500.063088 MHz
time domain size: 65536 points
width: 7507.51 Hz = 15.0131 ppm = 0.114555 Hz/pt
number of scans: 32

freq. of 0 ppm: 500.060000 MHz
processed size: 32768 complex points
LB: 0.300 GF: 0.0000

SpinWorks 4: 4-(3-(3,5-Dimethoxyphenyl)propenoyl) acetanilide

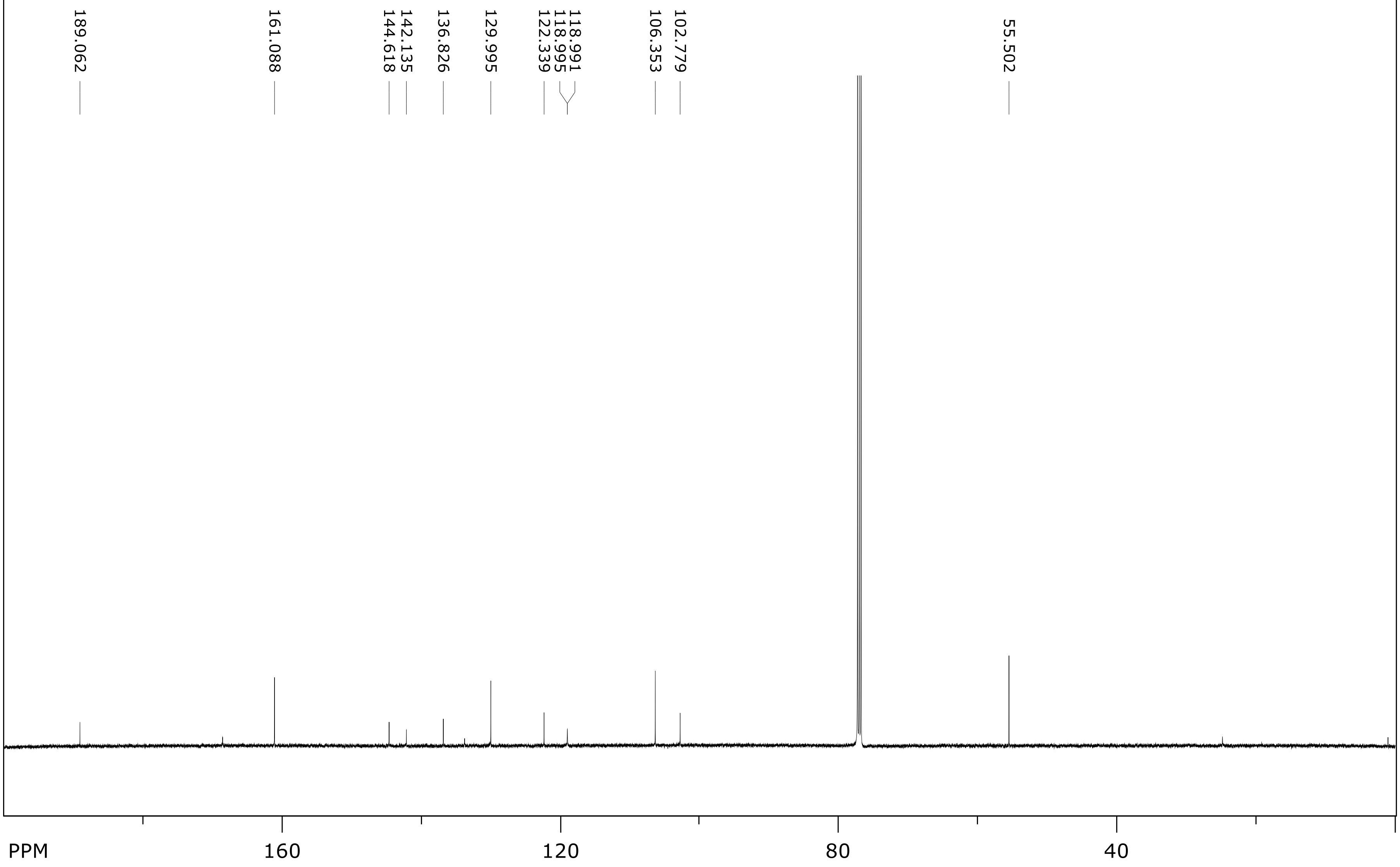

file: ...Data\EAV\500NMR\EAV-VIII-77C\1\fid expt: <zgpg>
transmitter freq.: 125.752761 MHz
time domain size: 262144 points
width: 26455.03 Hz = 210.3733 ppm = 0.100918 Hz/pt
number of scans: 1794

freq. of 0 ppm: 125.740187 MHz
processed size: 131072 complex points
LB: 1.000 GF: 0.0000
